# Pediatric arrowshot injury to cervical spinal cord-sagittal cord transection with no neurological deficit and good outcome: case report and review of literature

**DOI:** 10.1007/s00381-013-2095-7

**Published:** 2013-04-12

**Authors:** Tymon Skadorwa, Bogdan Ciszek

**Affiliations:** 1 Department of Pediatric Neurosurgery, Bogdanowicz Memorial Hospital for Children, 4/24 Nieklanska St., 03-924 Warsaw, Poland; 2 Department of Descriptive and Clinical Anatomy, Medical University of Warsaw, Warsaw, Poland

**Keywords:** Cervical spinal cord injury, Pediatric, Arrowshot

## Abstract

**Background:**

Penetrating spinal cord injuries (PSCI) in cervical region are extremely rare in children. They mostly occur in a mechanism of a gunshot or a stab injury with the use of sharp objects. Gunshot injuries are usually fatal or end up with tetraplegia. Stab wounds may be less severe and result in partial neurological syndrome. In the management of PSCI in children, reliable diagnostics and history of the patient are the most valuable for further decisions, which include early or delayed exploration either nonsurgical treatment. There exist no clear algorithm for antibiotic use in pediatric population—it depends on the site of an injury, presence of pathological secretion from the wound, and nature of the trauma. The use of steroids is controversial. The most common complications related to surgery include infections, edema, and hemorrhage. They may also be associated with the migration of small residual microtraumatizing agent. The literature lacks algorithms for management in children.

**Discussion:**

In this paper, an unusual case of almost total sagittal cervical cord transection is reported. The patient had no neurological symptoms and recovered with no complications. Diagnostic imaging on admission included X-ray and computed tomography. The patient underwent early surgical intervention with removal of foreign body from the cord and subsequent dural suturing. In the paper, the role of detailed history taking, adequate imaging, and drugs administration is discussed. The choice of distinct strategies is analyzed, and a revised literature review is presented in order to unify the management algorithm for pediatric PSCI.

## Background

Penetrating spinal cord injuries (PSCI) are relatively rarely observed effects of traumatic incidents. Stab injuries of the neck usually affect young adult males, but they seldom penetrate into the vertebral canal and affect the spinal cord [44, 56, 72]. The largest series of cases was reported from South Africa [50], where these incidents account for approximately 25 % of all back and spine injuries, while in the US population, they constitute up to 11 % of spine trauma [8]. Among PSCI reported in literature, low thoracic and lumbar level is dominating location, cauda equina is second the most commonly affected [5, 13]. Injuries to cervical spinal cord or craniocervical junction are very rare and reported as an unusual location which in most of cases leads to fatal prognosis [1, 14, 35, 52, 64]. Among all PSCI, gunshot mechanism is the most common and usually fatal [5, 10, 23, 27, 67, 71]. Stab wounds of the cervical spine are second most common incidents [36] and reported weapons are knives, screwdrivers, drill bits, or ice-picks [4, 53]. Stab wound in the neck has also been reported as an unusual suicide mechanism [28]. Among all published papers, pediatric population has poor but noticeable literature. Although penetrating injuries of the central nervous system in children have mostly orbito-cranial location [12, 22], cervical spine penetrations have also been reported [11, 39, 60]. Common penetrating agents are pencils, wooden splinters, bicycle spokes, and knives [33].

The symptoms of cervical PSCI mostly result directly from the trauma but may also be associated with vascular impairment, which was observed furthermore in autopsies [9, 68, 69]. Depending on the exact site of an injury, when not fatal, the most common neurological deficit includes high or low tetraplegia or end up with deep unilateral paresis [10]. When a cutting edge runs between the spinous and transverse processes (like in most of thoracic locations), it may result in Brown-Séquard syndrome [51, 57, 59], or radicular disorders, while running more laterally. The literature on this topic contains also descriptions of late consequences resulting from dislocation of residual microtraumatizing agents [29, 33, 66, 70].

## Case report

**Fig. 1:**
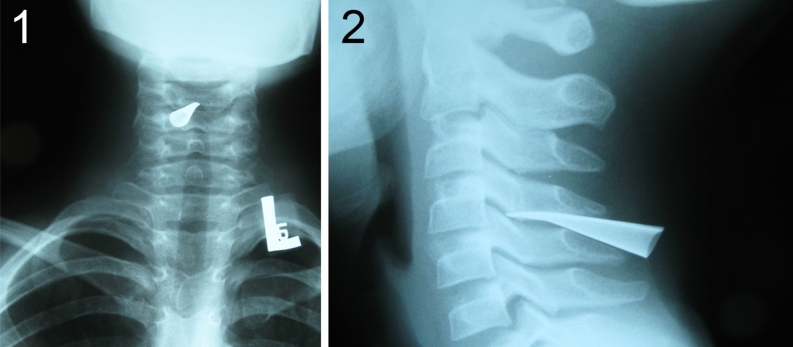
X-ray of the patient reveals the presence of residual metal arrowhead between C4 and C5 vertebra. *1* posteroanterior aspect; *2* lateral aspect

**Fig. 2:**
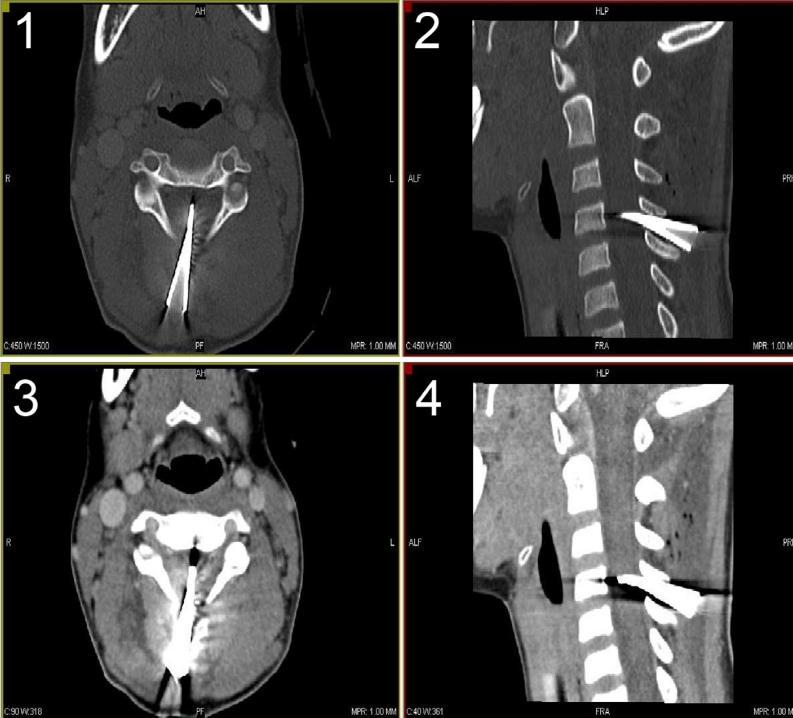
CT scans showing arrowhead stuck within the vertebral canal. *1*,*2* bone frame (axial and sagittal aspect); *3*,*4* nervous tissue frame (axial and sagittal aspect)

**Fig. 3:**
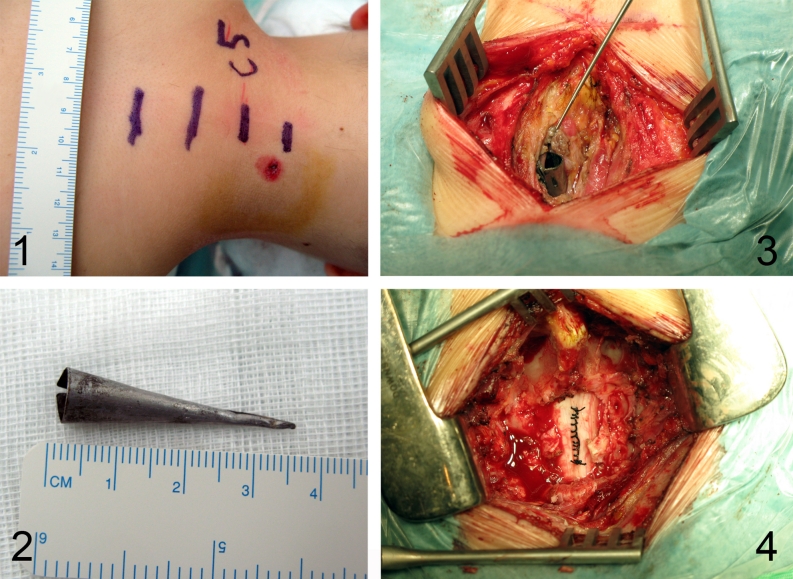
Intraoperative pictures. *1* location and size of the skin injury; *2* metal arrowhead removed from the spinal cord; *3* intraoperative aspect of an arrowhead residing in spinal cord; *4* dura with waterproof suture after removal of an arrowhead

**Fig. 4:**
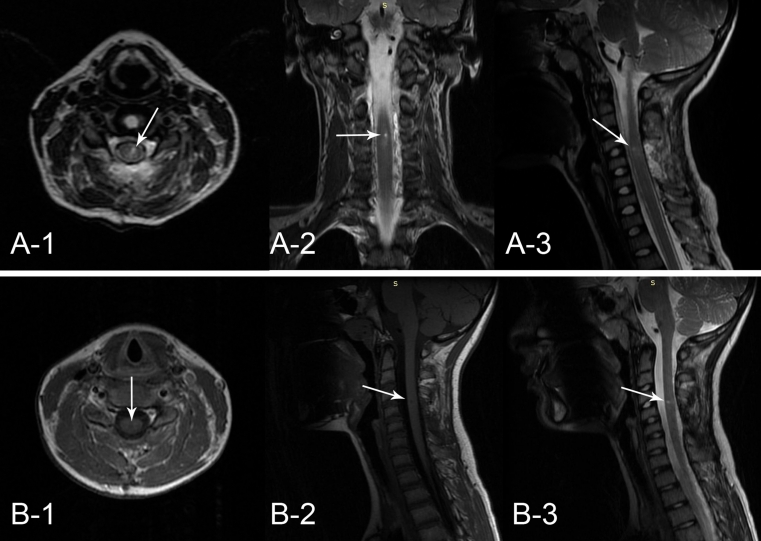
Postoperative control MRI scans. **a** early postoperative control; **b** control scans after 6 months from surgery. *1*,*2*,*3* different aspects. *Arrows* indicate the site of the lesion

Eleven-year-old boy presented to a hospital after he had been shot with an arrow by his younger brother while they were playing with a bow. The arrow stuck in a posterior surface of his neck (nuchal region) about the level of C5 vertebra and the arrow shaft was immediately removed by a brother. The parents brought their son to a local hospital where initial neck X-ray examination was performed. The only injury exposed to a physician’s view was a small, 1 cm long skin cut with no pathological secretion. The X-ray revealed a 35 mm long, cone-shaped metal arrowhead residing between C4 and C5 vertebra and protruding into the vertebral canal (Fig. 1). Nevertheless, the boy did not present any neurological deficit nor paraesthesia. He was equipped with a professional neck support, placed on a rescue board, and transmitted to Department of Pediatric Neurosurgery. On admission, he underwent head and neck CT examination, that showed the arrowhead stuck between arches of C4 and C5 vertebrae, protruding inside the vertebral canal and passing through the cervical spinal cord penetrating to 2/3 of its P-A dimension (Fig. 2). CT scans did not reveal any bone injury, intraspinal bleeding, nor edema. During neurological examination, the boy still did not demonstrate any neurological nor meningeal symptoms, numbness, neither sensation disorders. An initial treatment with methyl-prednisolone infusion according to National Acute Spinal Cord Injury Studies (NASCIS) protocol was implemented, and the patient was transported to an operating theater with a total of rescue supports. The boy was placed horizontally in prone position, with the nuchal region exposed. From the straight vertical skin incision, the C4-C5 area was achieved and the upper back muscles were carefully dissected and put aside. The exposed metal arrowhead was gently removed and no negative general patient reaction was observed. The arrowhead was cone-shaped and hollow, had 35 mm of length and 6 mm of diameter at its base (Fig. 3). Subsequently, a bilateral C4–C5 hemilaminectomy was performed in order to verify the injury of spinal cord and dura. In the traumatized area, no fresh bleeding was observed but the leakage of transparent cerebrospinal fluid. The dura was closed with continuous suture, and precise hemostasis of the operative field was performed. Finally, all layers were closed down. The patient was equipped with a neck support and spent next 2 weeks at the Department of Pediatric Neurosurgery. The treatment included continuous intravenous antibioticotherapy (ceftriaxone, amicacinum, and metronidazole) and lying in bed during first week of hospital stay followed by a gradual rehabilitation in the second. Postoperative neurological statement did not deteriorate; the boy did not suffer from paraesthesia nor other deficits. Postoperative wound healed properly. Control neck MRI, performed soon after, revealed no fresh bleeding nor edema in operated region. After 2 weeks, the treatment was terminated and the boy was discharged from the hospital in a state of complete health. Follow-up MRI examination after 6 months from surgery revealed no pathological collection nor postoperative spinal stenosis in operated region. The presence of focal (Ø up to 10 mm) hyperintensive region in the spinal cord did not influence his neurological statement (Fig. 4).

## Discussion and literature review

The example of our patient, such as many cases of head injury, exposes one of the aspects of pediatric neurotrauma: children often conceal the circumstances of the injury. In last few years of our experience, there have been a couple of cases with cranial penetrating injury, presented to our department with the small inlet wound previously stitched at the emergency room in their local hospitals. Subsequent interview in such cases commonly points to the falsification of the child’s description of the situation, which drives the diagnostics on the wrong track and makes the small wound on the head treated as a trivial injury, not requiring additional imaging tests. We treated patients in whom small wounds in the orbital region have sometimes even been overlooked in posttrauma physical examination. Neurological deterioration following penetrating spinal or cranial injury or change of the version of events by witnesses usually forces an extension of the diagnostics and radical change of management. In our opinion, pediatric medical professionals, especially the neurosurgeons, should show a maximum care to establish the true course of the events, because any delay in diagnostics can lead to intractable consequences, worsening the prognosis. In the case of our patient, the arrow shaft has been broken below the level of the skin, leaving only a small inlet wound. The boy’s parents were not witnesses of the accident. Therefore, the vigilance and inquisitiveness of the emergency doctor allowed to direct towards PSCI diagnosis.

The early management in PSCI cases includes immobilization and imaging [14]. Cervical X-ray seems to be an initial reference showing eventual foreign bodies stuck within the vertebral canal [51]. CT scanning facilitates an assessment of the range of an injury, shows the presence of residual broken bony fragments, and allows to track a possible trajectory of penetrating agent [49]. MRI is helpful in evaluation of the damage of the spinal cord [2, 25, 30, 42], its use is reduced, however, in case of residing metal objects.

Many centers take up early exploration when one of these conditions occurs: the presence of residual foreign body, any spinal compression, migration of small penetrating agent within the vertebral canal, acute neurological symptoms, or deterioration due to hemorrhage or the leakage of CSF [24, 37, 38, 55, 58, 62]. Remaining cases usually undergo conservative treatment with the use of antibiotics of large spectrum [31]. Early operative treatment of missile PSCI is debatable until migrating bullet provokes progression of neurological deficit. In our opinion, the open nature or unstable injury create indications to early operative treatment. Other cases should be treated conservatively with subsequent control imaging and antibiotic coverage. In severe penetrations, we use a triad of antibiotics: ceftriaxone, amicacinum, and metronidazole. The patient should be immobilized and remain in bed until the risk of bleeding or edema is reduced to minimum.

The role of methyl-prednisolone in early management of spinal injuries is still controversial. Bracken and Shephad (1997) recommend the use of steroids in blunt spinal injury according to NASCIS [7]. Some papers, however, postulate avoidance of steroids in penetrating spine injuries due to possible risk of immune compromise and subsequent infection [6, 21, 34]. In this case, we used steroids, according to Polish standards of medical practice and due to a fact that still there is no clear evidence of their adverse effects in PSCI in pediatric population.

The role of delayed operative inspection in PSCI is difficult to estimate—the literature is poor in cases of further removal of fragments stuck in the vertebral canal. Gupta et al. (2006) describe such a case in an adult with good outcome [18]. Other authors consider the indications to early and delayed exploration giving examples of effects of the presence of bony fragments or foreign agents retained within the vertebral canal [19, 29, 54, 66, 70].

Postoperative care should include the continuation of antibiotic cover, control early imaging, and immobilization of the patient. As for control imaging, CT may be more safe tool in detection of eventual retained metal fragments, especially in case of dural or spinal penetration. In our case, there was no doubt as to the completeness of the arrowhead removal. In case of bullets, however, that may break up to small, macroscopically invisible chips or flakes, this may be of great importance for further prognosis. We performed early control MRI scans in order to assess the range of spinal damage. Good clinical status of the patient allowed us to remove the collar. Late control MRI scanning was performed after 6 months of follow-up observation.

Many fundamental principles of management of neck penetrations, concerning specifically children, can be found in a reappraisal study, published by Mutabagani et al. in Journal of Pediatric Surgery (1995) [43]. The paper presents the largest pediatric series of cases (55 neck penetrations in 46 patients). The authors analyzed 33 male and 13 female patients in an average age of 9 years (range, 2–16). Thirteen injuries in the series involved neurological structures; however, only four of them were PSCI. The authors evaluated the symptomatology, demographic data, the choice of treatment strategy, and outcomes. They analyzed indications to wound exploration and undertook a discussion, which so far, has been based on the largest published experience. Therefore, an algorithm for the management of neck penetrations in children has been proposed by the authors. Despite the fact that it is very general and applies to all types of penetrating neck injuries, in our opinion, it pushes the model of management in the right direction, balancing the benefits of surgical treatment in relation to possible complications. Before World War II, most of penetrating neck injuries were treated nonoperatively with the mortality at the level up to 18 %. Early wound explorations, introduced during the war, decreased this rate to 7 % and led to the adoption of a rule of “mandatory operation” afterwards [3]. However, observed failure in operative treatment (33–76 % of negative explorations), resulting from the fact that some injuries might have been missed, led surgeons to change an approach and to propose better selection for operative treatment of penetrating neck injuries [43, 45, 46]. This idea is still present as far as spinal penetrations are concerned. So-called mandatory early exploration in PSCI cases seems unreasonable as some cases may benefit more from delayed intervention. In our opinion, the most problematic area in management is the decision on the timing of eventual surgery. The algorithm for management of PSCI in children should include guidelines concerning not only the eligibility for operative treatment, but also facilitating the choice between early and delayed intervention.

The complications of penetrating injuries of the spinal cord include, apart from neurological deficit, vast range of focal pathologies from which edematous and inflammatory processes directly follow the trauma [17]. Mella in 1967 give an example of meningitis as a result of an arrowshot [40]. The bleeding to vertebral canal was reported by Olshaker and Barish (1991). These authors described epidural collection following a stab wound to the neck with minimal symptomatology [47]. Harris in 2005 reports acute subdural hematoma as a result of a stab wound with Brown-Séquard syndrome in neurological examination [20]. Kuzelyi et al. (2001) describe the onset of diabetes insipidus as a consequence of thoracic spinal cord penetration [32]. As for cervical locations, vascular complications seem to be an important fact that should not be ignored in emergency diagnostics. Injury to vertebral artery due to stab wound may occur ipsi- or contralaterally; it may also result in cerebellar infarction [26, 48, 63]. Late consequences of cervical penetrating injury may include pathological processes in vertebral bodies [41]. Our patient did not present any direct neither delayed neurological deficit. In our opinion, only the injury running right through the middle of the spinal cord might have given such a result. The trajectory of an arrowhead have probably allowed to bypass larger vessels. Any further penetration, however, would most likely lead to tetraplegia or have a fatal end. Moreover, there was no certainty that these would not occur after operation. In our further analysis, the success of treatment depended on the coincidence of various facts: trajectory of the arrow, proper early management and timing, delicacy of the surgery, and appropriate postoperative care. Despite the high risk of infection, the boy did not develop any inflammatory process nor spinal edema. He did not require any rehabilitation afterwards. The only postoperative aspect to explain was the decision on whether to stabilize the cervical spine. Follow-up visits have shown the stability of this region of the neck, which, regarding young age of the patient and strong muscular apparatus, allowed to defer this decision.

Among various papers on PSCI, some report good recovery [15, 61] after more or less aggressive rehabilitation, especially in pediatric population [16, 65]. We did not noticed, however, any case of almost total spinal cord transection with no neurological deterioration and no delayed sequelae after operative treatment.

## Conclusions

Our experience confirms observations that the general and neurological statement of the PSCI patient may be very good on admission. Decision on operative or conservative treatment should be considered basing on the criterium of openness or instability of the injury. In all cases of cervical penetrations, however, after detailed diagnostic procedure, antibiotic coverage of large spectrum should be administered. In all cases, we check the anti-tetanus status of the patient, and a booster shot is implemented when needed. Operative exploration is undertaken in case of dural leak, local hemorrhage, residual foreign body, or dislocation of bony fragments. Every patient stays in bed with immobilization during the early postoperative period. Early control CT/MRI scanning is performed within 24–48 h from exploration and follow-up control after 3–6 months.

Children are specific group of patients due to blurred symptomatology and sudden, sometimes fulminant deterioration. The problem often appears at the very beginning—no clear report about what happened, resulting from child’s fear, handicaps the choice of appropriate procedure. Trauma, however, is often the only health problem of a child. Despite the turbulent course of a disease, children have also better prognosis. Therefore, detailed history with relation of witnesses (parents, siblings, and colleagues) and delicacy in management may be the most important asset in fast and reliable treatment of these difficult and demanding injuries.
